# Statin-loaded carbonated hydroxyapatite nanoemulsion controls matrix metalloproteinase-1 activity to enhance post-orthodontic stability in rats

**DOI:** 10.1590/1678-7765-2026-0023

**Published:** 2026-04-17

**Authors:** Ananto Ali Alhasyimi, Niswati Fathmah Rosyida, Pinandi Sri Pudyani

**Affiliations:** 1 Universitas Gadjah Mada Faculty of Dentistry Department of Orthodontics Yogyakarta Indonesia Universitas Gadjah Mada, Faculty of Dentistry, Department of Orthodontics, Yogyakarta, Indonesia; 2 Universitas Gadjah Mada Center of Excellence for Carbonate Apatite-based Extracellular Matrix and Adjuvant Indonesia Universitas Gadjah Mada, Center of Excellence for Carbonate Apatite-based Extracellular Matrix and Adjuvant, Indonesia

**Keywords:** Carbonated hydroxyapatite, Matrix metalloproteinase, Nanoemulsion, Relapse, Simvastatin

## Abstract

**Background:**

The persistence of periodontal ligament (PDL) and alveolar bone remodeling following appliance removal is a key factor in orthodontic relapse, which is a significant barrier to treatment stability. In this study, a statin-loaded carbonated hydroxyapatite (CHA) nanoemulsion was evaluated as a local therapy to control relapse rate by modulating matrix metalloproteinase-1 (MMP-1) in rats.

**Methodology:**

A total of 48 rats (n=48) were allocated to control, simvastatin, CHA hydrogel, or CHA–simvastatin nanoemulsion groups. Treatments were delivered intrasulcularly during stabilization, and relapse was measured at days zero, one, seven, and 14 post-debonding. MMP-1 expression in the PDL was assessed using immunofluorescence at the same time points. Collected data were evaluated using analysis of variance.

**Results:**

Relapse occurred in all groups, with the highest relapse rate during early observation. At day one, the relapse rate was significantly reduced in treatment groups compared with the control, and the CHA–simvastatin group showed the lowest relapse rate (control 1048±20.20 vs. CHA–simvastatin 549.33±27.22 μmd^−1^; p=0.001). MMP-1 expression peaked at day one and was significantly lower in all treatment groups than control, with the greatest suppression in the CHA–simvastatin group (16.33±2.08 vs. 7.33±0.57 positive cells/field; p=0.001). By day 14, relapse rates and MMP-1 expression decreased across groups, with persisting advantages for the combined treatment.

**Conclusion:**

Local CHA-simvastatin nanoemulsion decreased early relapse and downregulated MMP-1 expression in rats, suggesting a promising adjunctive strategy to enhance post-orthodontic stability.

## Graphical abstract

**Figure:**
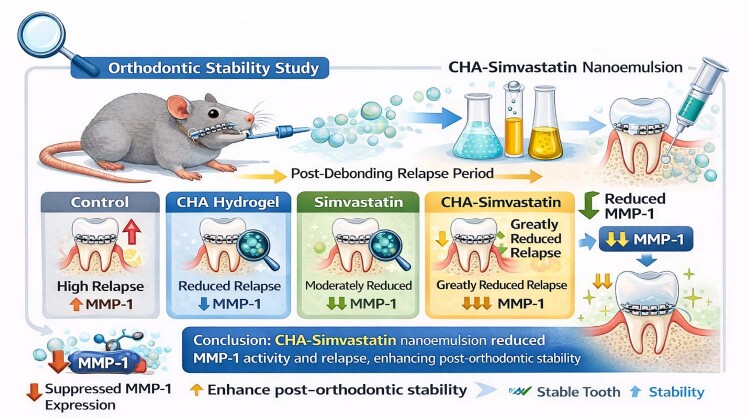

## Introduction

Orthodontic treatment aims to achieve and preserve an ideal occlusion, defined by harmoniously functioning teeth and facial structure. The subsequent disruptions in the local mechanical environment resulting from orthodontic appliances removal may lead to the teeth shifting from their corrected positions.^1^ Orthodontic relapse is the term used to describe the unavoidable and unfavorable movement of teeth following active orthodontic tooth movement (OTM). This phenomenon continues to be one of the most significant obstacles in orthodontics.^2^ Relapse rates continue to be exceptionally high, despite the implementation of effective retention protocols, meticulous treatment planning, and precise diagnosis. Data suggest that the incidence of relapse following orthodontic treatment ranges from 70% to 90%.^3^ A recent study reveals that ongoing periodontal ligament (PDL) and bone remodeling, along with active osteoclast activity, contribute to relapse.^4^ According to subsequent histological investigations, the tissue and molecular reaction patterns that are promptly observed following the interruption of orthodontic force are identical to those observed during OTM.^4,5^

OTM is facilitated by the continuous resorption of alveolar bone on the compression side of the tooth, which is mediated by osteoclasts, and the formation of new bone on the tension side by osteoblasts, a phenomenon known as periodontal remodeling.^6^ PDL is a tissue remarkably responsive to mechanical forces and connects the tooth to the adjacent alveolar bone. The tendency of teeth to migrate through the bone depends on the PDL, which is an exceptionally mechanoresponsive tissue. Mechanical forces are essential for sustaining periodontal homeostasis by triggering an integrated reaction across all PDL components. This encompasses intricate mechanisms including osteoimmunology, cellular communication, mechanotransduction, and modifications of the extracellular matrix (ECM).^7^ The releases of the cytokines interleukin-1β and tumor necrosis factor-α in the PDL promote osteoclast differentiation, activity, and viability, while simultaneously elevating inflammation and matrix metalloprotease (MMP) levels.^8,9^ Cathepsins and MMPs, particularly collagenase, breakdown the PDL ECM and boney organic matrix, enabling osteoclasts attachment for bone resorption.^10^ Synthetic MMP inhibitors have been proven to reduce OTM.^11^ Bildt, et al.^12^ (2009) demonstrate that MMP-1 is implicated in enhanced remodeling of the PDL and alveolar bone during OTM. Consequently, inhibiting MMP-1 expression may serve as a strategy to avert orthodontic relapse.

Relapse occurs by the same biochemical mechanism as OTM, except in the reverse order. The resemblance in physiological patterns among OTM and relapse indicates that pharmacological therapies aimed at the molecular pathways involved in PDL and alveolar bone remodeling may effectively reduce orthodontic relapse. Tissue engineering approaches have been suggested to improve tooth stability and avert orthodontic relapse.^13^ Carbonate apatite (CHA) can regulate calcium release and facilitate bone regeneration, rendering it an appropriate material for bone tissue engineering. Furthermore, CHA may serve as a mechanism for tailored drug delivery.^14^ Simvastatin has lately been examined for its potential efficacy in treating osteoporosis via enhancement of bone regeneration. The systemic treatment of simvastatin has demonstrated the ability to suppress osteoclastic bone resorption and promote bone growth, presenting a viable solution for mitigating post-orthodontic relapse. Its enduring safety profile renders it an appropriate choice for incorporation into orthodontic procedures.^1^ Nonetheless, elevated amounts of simvastatin necessary for skeletal effects may result in toxicity. Local delivery of nanoemulsions has emerged as a viable approach to reduce hepatotoxicity and improve efficacy in facilitating bone healing.^15,16^ This study investigates whether the administration of nanoemulsion CHA-simvastatin can regulate MMP-1 expressions thereby ameliorating post-orthodontic relapse rate in rats.

## Methodology

### Preparation of nanomaterials and hydrogel carrier

The experimental procedure commenced with the preparation of the CHA hydrogel. This preparation required the combination of type-I gelatin, distilled water, and sodium citrate to produce a fundamental solution. After the addition of calcium hydroxide, the resultant mixture was magnetically stirred for one hour. The resultant hydrogel was subsequently submitted to treatment for one hour to facilitate the requisite electrostatic binding. The CHA was mixed with distilled water to create the standard CHA hydrogel.

The standard CHA hydrogel was produced by combining the CHA with distilled water. The simvastatin nanoemulsion gel was created by combining oleic acid, Tween 80, simvastatin, and distilled water with a gel matrix that was equivalently composed of carbomer and heated water. Prior to synthesis, the statin nanoemulsion was combined with CHA to generate a solution. Simvastatin nanoemulsion (0.05 mg/10 μL) was combined with CHA (0.1 mg/10 μL) in a 1:1 ratio to produce the CHA-simvastatin nanoemulsion gel.

### Animal handling and experimental design

The study used 48 eight-week-old male *Sprague–Dawley* rats (250±25 g). Animals were housed at 20–25°C under controlled humidity and had free access to standard ground food and water throughout the study. All procedures were conducted in accordance with the ARRIVE guidelines. Ethical approval was obtained from the Ethics Committee of the Faculty of Dentistry, UGM, with approval number 41/UN1/KEP/FKG-RSGM/EC/2023.

Prior to commencement, the animals were submitted to a one-week acclimatization period to facilitate their adaptation to the laboratory environment and dietary regimen. Group C was the control group, Group St was the statin nanoemulsion group, Group CHA was the CHA hydrogel group, and Group CHA-St was the CHA-statin nanoemulsion combination. The subjects were randomly designated serial numbers and subsequently assigned to one of four groups: Group C, Group St, Group CHA. The groupings were subsequently allocated in a fair manner. From each major group, a random subset of four subgroups was generated based on the observation days (zero, one, seven, and 14 days). All experimental procedures were executed under general anesthesia to optimize accessibility and minimize physiological distress. Anesthesia was induced intramuscularly using Ketamine (35 mg/kg; Woerden, The Netherlands), and xylazine (5 mg/kg; ADWA, Egypt).

**Figure 1:**
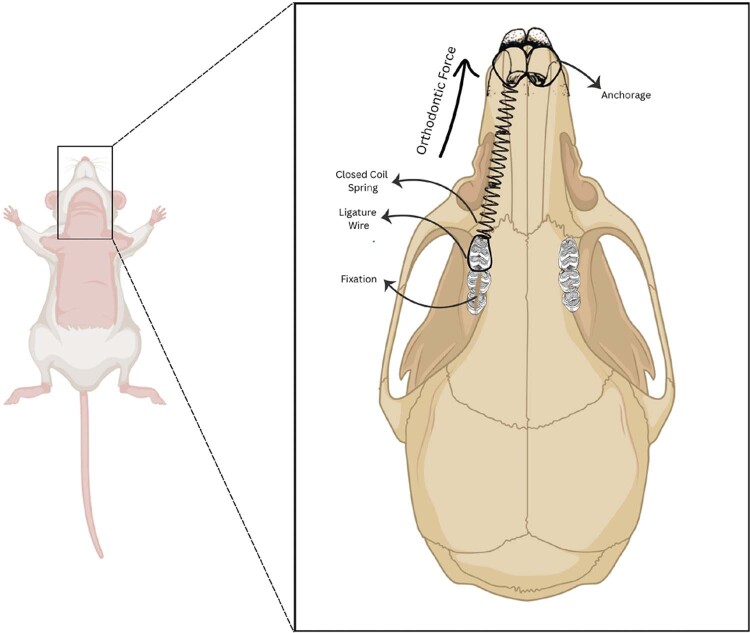
Orthodontic appliance installation. A closed-coil nickel-titanium spring is placed between the right maxillary first molar and incisors using a ligature wire. The device induces mesial movement (30 g force) of the first molar.

A short, closed nickel-titanium coil spring (1 mm in diameter, 0.14 mm in thickness, and 6 mm in length; Ormco, USA) was attached after anesthesia using a split-mouth design. During installation, one end of the spring was fastened between the right maxillary first molar and the incisor. To make it easier to use the ligature wire, the other end of the spring was attached to the maxillary incisor after grooves were made in both incisors with a spherical drill. A 0.09 mm ligature wire was wrapped around the first molar and threaded between the second and third upper molars. A comparable ligature wire was fashioned in a figure-eight configuration encircling the incisors and tightened with artery forceps until it conformed to the fissures. The orthodontic device induced mesial movement (30 grams force) of the first molar (Figure 1). To neutralize the force and transition the device into a retainer, the closed-coil spring was blocked with composites. This commenced the seven-day stabilization period, during which the inter-molar spacing was maintained at ± 3 mm. After the stability period concluded and the appliances were removed, molar relapse movement commenced.

The treatment groups were given their respective doses (15 μL of CHA hydrogel, statin nanoemulsion, or the CHA-St nanoemulsion) on days zero, four, and seven during the stabilization phase. The treatment was administered through an intrasulcular route, directly into the distal part of the gingival sulcus of the incisors, while the subjects were under general anesthesia.

### Measurement of relapse rate

The distance from the mesial end of the maxillary second molar to the distal end of the maxillary first molar, indicative of the relapse distance, was measured on days zero, one, seven, and 14 post-debonding of the appliances utilizing a digital caliper (Pro-Max®, China) with a minimum measurable increment of 0.01 mm. The distance was assessed twice, immediately post-debonding (D0) and on the day of sacrifice (D1), with the relapse distance computed as D2 = D0 − D1.

The relapse distance data were utilized to compute the relapse rate (micrometers per day/μmd^1^), representing the extent of relapse movement per day. All measurements were conducted by two experienced researchers who were blinded to the administered regimen and repeated three times. The average of these values was utilized as the representative value for each distance.

### Immunofluorescence staining of matrix metalloproteinases

The animals in each group were euthanized using an overdose of ketamine and xylazine at zero, one, seven, and 14 days following debonding. After euthanasia, they were decapitated, and their maxillary bones were carefully extracted. The retrieved tissue was immersed in a 10% formalin solution for 24 hours. The specimens were then demineralized in a 10% ethylene diamine tetraacetate (pH 7.4) solution at 25°C for six weeks. After embedding each specimen in paraffin wax, a microtome was used to cut 4-μm thick slices at 50-μm intervals from the cervicoapical two-thirds region.

The tissue sections were submitted to deparaffinization in xylene for 15 minutes, repeated twice, to remove paraffin wax. Subsequently, the sections were hydrated by a series of alcohol washes: 100% alcohol for five minutes (twice), followed by one minute each of 90%, 80%, and 70% alcohol concentrations. The elements were then washed with distilled water for three minutes to ensure sufficient hydration. After deparaffinization, antigen retrieval was performed by incubating the sections at 95°C for 15 minutes using a slow cooking method. Following the antigen retrieval phase, the sections were washed three times with PBS for five minutes each.

The tissue slices were then incubated with the primary antibody (anti-MMP1, FineTest®, FNab05233) at the specified dilution overnight at 4°C. The prolonged incubation enhances the specific binding of the primary antibody to its target antigen on the tissue sections. After incubation with the primary antibody, the sections underwent three five-minute washes with PBS to remove any unbound antibody. The subsequent stage involved incubation with a fluorochrome-conjugated secondary antibody (Abbkine Scientific Co., KTD107-EN) for one hour at 25°C. The primary antibody's species was determined by selecting the secondary antibody, which was then conjugated to a fluorochrome for visualization under a fluorescence microscope.

The sections were washed twice with PBS for three minutes each on a shaker before being incubated with the secondary antibody. DAPI (4’,6-diamidino-2-phenylindole) was applied at a 1:1000 dilution to stain cell nuclei for 10 minutes at room temperature. During the incubation period, an adequate concentration of DAPI solution (300 nM) was utilized to ensure comprehensive coverage of the cells. Immediately after DAPI staining, the transparencies were rinsed with PBS three times, with each treatment lasting five minutes. Subsequently, the slides were mounted with the appropriate medium. To identify the specific fluorescence signals indicative of MMP-1 in the tissue sections (green fluorescence), the affixed slides were subsequently examined using a fluorescence microscope. The anticipated outcome will be the identification of specific fluorescence that is associated with the target proteins (MMP-1). The target molecule's position is indicated by this green signal, which also serves to indicate the cellular architecture and functions.

### Statistical analysis

This study employed a two-way analysis of variance (ANOVA) to determine group interactions and differences in the data analysis. We subsequently employed Tukey’s honest significant difference test to identify statistically significant differences among groups. A significant difference was established with a *p* value of less than 0.05. The Statistical Package for Social Sciences, version 25 (IBM, USA), was used in analytical procedures.

## Results

All animals showed good tolerance to the experimental approaches, and no discernible detrimental effects were noted during the study period. There were orthodontic relapses in all groups after appliance removal. In all experimental groups, relapse climbed rapidly during the initial observation phase and subsequently diminished over time. Statistical analysis indicated substantial differences in relapse rates across the experimental groups at day zero, day one, day seven, and day 14 (*p*<0.05).

**Figure 2:**
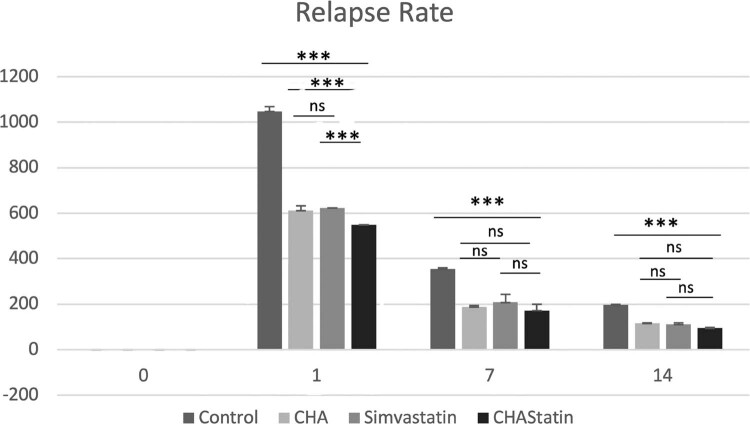
Relapse rate during the post-orthodontic relapse period. Bar graph showing relapse rate at day zero, day one, day seven, and day 14 after orthodontic appliance removal in the control, CHA, simvastatin, and CHA-simvastatin groups. Data are shown as mean ± standard deviation. Statistical analysis was performed using one-way ANOVA followed by Tukey’s HSD post hoc test. Asterisks indicate statistically significant differences between groups (*P<0.05; **P<0.01; ***P<0.001), while ns denotes no statistically significant difference.

Day zero marks the immediate post-debonding phase and indicates the initial quantifiable relapse dynamics. On day one, the control group demonstrated the greatest relapse rate, which is a marked early relapse after appliance removal. Conversely, markedly reduced relapse rates were noted in the CHA, simvastatin, and CHA–simvastatin in comparison to the control group (*p*<0.001). The post hoc analysis revealed that the CHA–simvastatin group had a significantly lower relapse rate compared to both the CHA and simvastatin groups at this time point (*p*<0.001). On day seven, rates of relapse were significantly higher in the control group relative to all treatment groups (*p*<0.001). No statistically significant differences were seen among the CHA, simvastatin, and CHA–simvastatin groups (*p*>0.05), indicating a convergence of treatment effects during the intermediate period of post-orthodontic remodeling. By day 14, relapse rates had diminished significantly across all groups. The control group had a considerably higher relapse rate compared to the CHA–simvastatin group (*p*<0.001). No significant differences were noted between the CHA and CHA–simvastatin groups or between the simvastatin and CHA–simvastatin groups (*p*>0.05), suggesting a reduction in intergroup differences as the remodeling process advanced (Table 1, Figure 2).

**Table 1:** Descriptive values and ANOVA-test comparisons of relapse rate (micrometers per day/μmd−1) measurementsa

Parameter	Control group (A)	CHA(B)	Simvastatin (C)	CHA-Statin group (D)	*P*- value	*Post hoc* comparison
**Day 0**	-	-	-	-	-	-
Day 1	1048±20.20	611.67±20.82	623.33±35.11	549.33±27.22	0.001*	A>B<C>D
Day 7	354.76±4.36	187.62±6.43	207.61±7.04	172.37±3.59	0.001*	A>B<C>D
Day 14	196.14±2.89	115.47±2.50	110.95±3.93	94.07±2.13	0.001*	A>B=C=D

Values are shown as mean ± standard deviation

^a^ Tested by two-way analysis of variance, and *post-hoc* Tukey HSD analysis test;

* *P*<.05, Significant differences between groups

**Figure 3:**
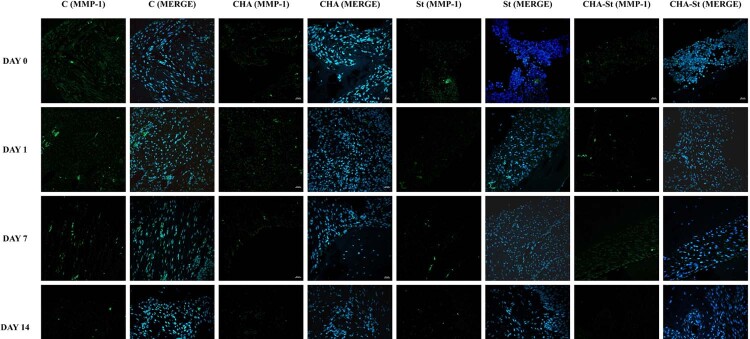
Compound panel displaying immunohistochemical results on samples labeled with antibody against MMP-1. The staining pattern of this protein shows a distinctly detectable fluorescence within the periodontal ligament. The blue fluorescence (MERGE) indicates nuclear staining with DAPI, revealing the presence of the cells.

**Figure 4:**
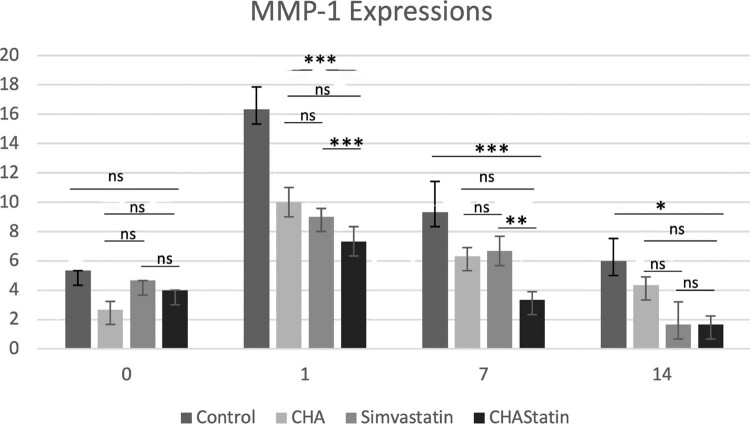
MMP-1 expression during the post-orthodontic relapse period. Bar graph showing the mean number of MMP-1–positive cells per field at day zero, day one, day seven, and day 14 after orthodontic appliance removal in the control, CHA, simvastatin, and CHA-simvastatin groups. Data are shown as mean ± standard deviation. Statistical analysis was performed using one-way ANOVA followed by Tukey’s HSD post hoc test. Asterisks indicate statistically significant differences between groups (*P<0.05; **P<0.01; ***P<0.001), while ns denotes no statistically significant difference.

Immunofluorescence reaction performed on PDL showed a clearly detectable staining pattern for MMP-1 during the relapse phase (Figure 3). In all images, the DAPI staining (blue channel) confirmed the presence of MMP-1 in cells. On day zero, MMP-1 expression was low fluorescence and consistent throughout all experimental groups, with no statistically significant changes observed among the control, CHA, simvastatin, and CHA–simvastatin groups (*p*>0.05), indicating an equivalent baseline condition immediately following appliance removal. MMP-1 expression significantly increased in all groups on the first day. The control group had the highest level of MMP-1 expression (16.33±2.08 positive cells/field). Nevertheless, the CHA (10.00±1.02 positive cells/field), simvastatin (9.00 ± 1.00 positive cells/field), and CHA–simvastatin (7.33±0.57 positive cells/field) groups showed a significantly lower MMP-1 expression than the control group (*p*<0.05). A graded reduction in MMP-1 expression was noted in the sequence of control > CHA > simvastatin > CHA–simvastatin. On day seven, MMP-1 expression decreased across all groups but was significantly elevated in the control group (9.33±1.52 positive cells/field) compared to the CHA (6.33±0.57 positive cells/field), simvastatin (6.67±1.53 positive cells/field), and CHA–simvastatin (3.33±0.57 positive cells/field) groups (*p*<0.05). Among the treatment groups, the CHA–simvastatin group consistently showed the lowest MMP-1 expression. By day 14, MMP-1 expression diminished across all groups. The simvastatin and CHA–simvastatin groups had the lowest and comparable levels of MMP-1 expression (both 1.67±0.57 positive cells/field), significantly lower than those recorded in the control and CHA groups (*p*<0.05). No statistically significant difference was observed between the simvastatin and CHA–simvastatin groups at this time point (*p*>0.05; Table 2, Figure 4).

**Table 2:** Descriptive values and ANOVA-test comparisons of MMP-1 expression (MMP-1-positive cells/field) measurementsa

Parameter	Control group (A)	CHA (B)	Simvastatin (C)	CHA-Statin group (D)	*P*- value	*Post hoc* comparison
Day 0	5.33±1.53	2.66±0.57	4.66±0.57	4.00±1.00	0.193	
Day 1	16.33±2.08	10.00±1.02	9.00±1.00	7.33±0.57	0.001*	A>B>C>D
Day 7	9.33±1.52	6.33±0.57	6.67±1.53	3.33±0.57	0.001*	A>C>B>D
Day 14	6.00±1.00	4.33±0.57	1.67±0.57	1.67±0.57	0.001*	A>B>C=D

Values are shown as mean ± standard deviation

Tested by two-way analysis of variance, and *post-hoc* Tukey HSD analysis test;

* *P*<.05, Significant differences between groups

## Discussion

Orthodontic relapse remains a major concern, frequently jeopardizing long-term treatment success and patient satisfaction. Relapse maintains a persistent clinical challenge due to continuing biological processes that facilitate OTM, which can often proceed in the opposite direction following appliance removal.^17^ In this study, relapse occurred across all groups, with the highest rate observed initially, followed by a gradual decline over time. Importantly, the local administration of CHA hydrogel, simvastatin, and particularly CHA–simvastatin nanoemulsion markedly decreased the relapse rate compared to the control at various observation points, with the most pronounced difference observed on day one. The “early burst” of relapse is biologically plausible because the immediate post-debonding phase involves rapid reorganization of periodontal fibers and acute inflammatory signaling, both of which accelerate ECM turnover. This trend aligns favorably with previous studies, which demonstrated that the orthodontic relapse commenced rapidly after appliance removal, followed by a gradual reduction in relapse.^4,5^ The elevated relapse rate was observed promptly after appliance removal, and it gradually diminished over the course of the subsequent 21 days.^2^ This phenomenon can be elucidated by a theory suggesting that apoptosis of vascular cells and reduced angiogenesis occur within several days following appliance removal.^4,5^ Furthermore, even seven days after orthodontic relapse, chronic inflammatory cells persist in partially occupying the periodontal environment; however, physiological periodontal turnover gradually reestablishes itself by day 14.^18^ Consistent with this, our findings also indicate that by day 14, the relapse rates were very low but remained stable, suggesting that periodontal homeostasis had largely been reestablished and the biological drive for relapse had substantially diminished.

In the results, the control group showed the highest relapse rate at day one, whereas the CHA–simvastatin group showed the lowest, suggesting that the combined formulation is particularly effective during the early, high-turnover phase of PDL remodeling. CHA promotes remodeling by elevating calcium and phosphate concentrations in the local milieu, which supports new bone synthesis and modulates osteoblast activity.^19^ Furthermore, elevated extracellular calcium concentrations promote DNA synthesis and chemotaxis in osteoblastic cells while inhibiting osteoclastic formation, hence potentially preventing orthodontic relapse.^2,20^ In parallel, statins can influence osteoclastogenesis by shifting the RANKL/OPG balance, thereby limiting bone resorption that contributes to relapse progression.^1^ The local delivery of simvastatin, encapsulated in nanocarriers, may address toxicity associated with systemic administration, enabling targeted delivery that maximizes therapeutic outcomes.^21^

Relapse transpires through the same biochemical mechanism as OTM; therefore, a delicate and precisely balanced ECM remodeling process within the periodontium is essential while preserving periodontal functional integrity.^2,22^ PDL cells' increased or decreased expression of particular mRNA, as well as protein synthesis, are thought to play important roles in their adaptive responses to various types of mechanical stress.^23^ MMPs, which have been implicated in the remodeling of bone and connective tissues, appear to be engaged in PDL remodeling during orthodontic movement. Matrix metalloproteinases, or MMPs, are zinc-dependent ECM enzymes capable of degrading enzymes that have been proven to play a crucial role in ECM regeneration.^24^ MMP-1, a key enzyme involved in the breakdown of collagen, plays a crucial role in the remodeling of ECM during OTM. In the context of orthodontic relapse, MMP-1 activity may be involved in bone remodeling after appliance removal, potentially contributing to the loss of tooth stability. Garlet, et al.^25^ (2007) discovered considerably increased MMP-1 expression levels in PDL tissue at both tension and compression sites, with a significantly greater expression level at the compression zone. This suggests a greater role for MMP-driven ECM protein breakdown at the compression site. By regulating MMP-1 activity, it may be possible to control the extent of collagen degradation and thereby reduce the risk of relapse. Here, MMP-1 expression increased sharply at day one in all groups, but treatment groups had significantly lower MMP-1 expression than control, with the greatest suppression in the CHA–simvastatin group. This graded reduction (control > CHA > simvastatin > CHA–simvastatin) supports the hypothesis that the formulation enhances post-orthodontic stability by suppressing collagenolytic activity. The results are consistent with the findings of Cantarella, et al.^26^ (2006), indicating that MMP-1 expression levels are prominent during the initial phases of tooth movement.

Notably, by day 14, MMP-1 expression decreased across all groups, and simvastatin and CHA–simvastatin reached similarly low levels, consistent with a transition from an acute remodeling environment toward physiologic turnover. This may explain why intergroup differences tend to narrow at later time points: as baseline remodeling re-establishes, the “ceiling” for further MMP-1 suppression (and thus treatment separation) becomes smaller. Beyond lipid-lowering, statins have pleiotropic effects relevant to periodontal healing and bone remodeling.

Mechanistically, statins downregulate MMP transcription by inhibiting both NF-κB and activator protein-1 (AP-1), which may help preserve periodontal ligament integrity and limit soft tissue breakdown.^27,28^ This is consistent with the reduced MMP activity reported and provides a rationale for the decreased MMP-1 signal observed in treated PDL. CHA is an osteoconductive biomaterial that not only facilitates bone formation but also provides a potentially superior structural delivery system with targeted and sustained drug release capabilities.^29^ Whereas nanoemulsion as an innovative drug delivery system shows optimistic solutions by improving drug solubility, stability, and targeted delivery. Due to their extremely small size, which facilitate enhanced absorption and precise delivery of therapeutic agents, and their capability to regulate drug release, nanoemulsions show significant potential for advancing the treatment of oral diseases. Their versatility as delivery systems renders them suitable for a range of applications, including topical administration in dentistry.^30^ These findings support the concept that targeting ECM degradation and inflammatory remodeling, while simultaneously providing a pro-regenerative mineral environment, may improve post-orthodontic stability and potentially reduce dependence on prolonged mechanical retention.

Combining CHA with simvastatin in a nanoemulsion-hydrogel system plausibly provides (1) improved local retention and controlled release and (2) suppression of destructive remodeling (via MMP-1/inflammation control). This dual action is consistent with the early reduction in relapse rate and the consistently lowest MMP-1 expression in the CHA–simvastatin group at days one and seven. However, the study's limitations include a short observation period, a limited sample size, and an emphasis on a single treatment method. Future research should incorporate human clinical trials, larger sample sizes, and extended follow-up periods to verify the findings' relevance to humans.

## Conclusion

Local administration of CHA–simvastatin nanoemulsion reduced post-orthodontic relapse rate and suppressed MMP-1 expression in rat PDL, with the greatest effect observed during the early post-debonding phase.
